# Early Taurine Administration Decreases the Levels of Receptor-Interacting Serine/Threonine Protein Kinase 1 in the Duchenne Mouse Model *mdx*

**DOI:** 10.3390/brainsci15111175

**Published:** 2025-10-30

**Authors:** Marthe Dias, Hanne Dhuyvetter, Ella Byttebier, Caroline Merckx, Jan L. De Bleecker, Boel De Paepe

**Affiliations:** 1 Department of Neurology, Ghent University, Corneel Heymanslaan 10, 9000 Ghent, Belgium; marthe.dias@ugent.be (M.D.);; 2 Neuromuscular Reference Center, Ghent University Hospital, Corneel Heymanslaan 10, 9000 Ghent, Belgium

**Keywords:** Duchenne muscular dystrophy, *mdx*, taurine, osmolyte, receptor-interacting Serine/Threonine protein kinase 1

## Abstract

**Background/Objectives**: The progressive life-limiting disorder Duchenne muscular dystrophy (DMD) arises from the absence of dystrophin protein at the muscle cell membrane, which leads to progressive contraction-induced damage. Despite the advancements in molecular therapies aimed at reintroducing (partially functional) dystrophin in patients, a cure for DMD remains elusive. Taurine supplements have been proposed as a potential supportive treatment for DMD, based upon encouraging results in the mouse model *mdx*. **Methods**: In a previous study, we observed improvements in skeletal muscle histology and a reduction in the expression of inflammatory markers after short-term treatment with 4.6 g taurine per kg body weight during the initial stages of the disease. In this follow-up study, we examined cell death and tissue restoration protein levels in *mdx* subjected to the same treatment regimen, utilizing proteome arrays, Western blotting, and immunofluorescence. **Results**: We report that, while the levels of apoptotic and autophagic proteins remained constant, selective and significant decrease in receptor-interacting Serine/Threonine protein kinase 1 (RIP1) levels could be observed in taurine-treated *mdx* compared to untreated *mdx*. RIP1 was immunolocalized to muscle fibers, with faint homogeneous staining in age-matched healthy controls shifting to a heterogeneous staining pattern in *mdx*, the latter diminishing with taurine treatment. **Conclusions**: Given its role as a molecular switch in cell fate decisions, the observed taurine-induced downregulation of RIP1 supports the potential beneficial effects of the osmolyte in *mdx*.

## 1. Introduction

Duchenne muscular dystrophy (DMD) is an X-linked genetic disorder caused by mutations in the *DMD* gene, which results in the absence of the structural protein dystrophin and the dismantling of the dystrophin-associated oligomeric protein complex at the sarcolemma. DMD patients accumulate contraction-induced damage to muscle fibers, causing progressive muscle wasting and weakness that typically manifest between the ages of two and five years old. The absence of functional dystrophin triggers a cascade of events that provokes chronic inflammation and disfavors muscle regeneration [1], leading to the replacement of muscle cells with connective and fatty tissue [2]. An essential and early pathogenic mechanism that aggravates DMD is metabolic dysregulation, which includes mitochondrial dysfunction. Without dystrophin to stabilize the membrane, tears in muscle cells allow calcium ions to enter uncontrollably, resulting in cellular catabolism and mitochondrial signaling of apoptotic cell death [3]. Dysfunction of the mitochondrial tricarboxylic acid cycle [4] and of oxidative phosphorylation [5] lead to a reduced supply of reducing equivalents and energetic failure. Skeletal muscle’s high energy requirements make this tissue strongly and progressively affected in DMD patients. Subsequently, patients encounter respiratory and cardiac complications, which culminate in early death most often before the age of 40 years.

While there is currently no cure for DMD, significant research efforts have resulted in the recent clinical approval of molecular therapies that aim to introduce partially functional dystrophin, recapitulating a less severe Becker muscular dystrophy-like phenotype [6]. Gene therapy has become available that delivers a shorter version of the *DMD* gene packaged into a recombinant adeno-associated virus (AAV) capsid [7]. In antisense oligonucleotide (ASO)-based therapy, dystrophin pre-mRNA complementarity of short synthetic nucleic acid sequences leads to the selective skipping of destabilizing *DMD* exons thereby restoring the reading frame, again leading to a shorter version of the dystrophin protein [8]. A notable additional constraint of ASO interventions is their *DMD* mutation specificity, limiting their efficacy to a subset of patients only. Consequently, the development of novel and supportive therapies for DMD remains of paramount importance today.

In recent years, taurine (2-aminoethanesulfonic acid) has been posited as an amenable supportive treatment for DMD. The non-protein-building amino acid is a naturally occurring derivative of cysteine with osmo-protective properties of which levels are particularly high in skeletal muscle. The main sources of taurine are foods such as meat, seafood, and dairy products. In addition to intake through the diet, small quantities of taurine are synthesized in the liver through amino acid conversion. During periods of illness and stress, taurine becomes essential, hence its classification as a conditionally essential amino acid. The importance of taurine for our health is illustrated by deficiencies causing retinal disorders [9] and accelerated aging [10]. The potential muscle-fortifying properties of taurine have been a subject of scientific inquiry, with studies suggesting a stimulatory effect on exercise performance; however, opinions diverge regarding its efficacy to mitigate muscle damage. While taurine has been reported to reverse oxidative damage and restore muscle function in overused muscles [11], further research is necessary to improve our understanding of the mechanisms involved [12]. Taurine supplements are currently being investigated in DMD animal models, with decreased muscle damage and mitigated expression of inflammatory and oxidative stress markers reported in the standard mouse model *mdx* [13,14,15,16]. However, researchers also observed undesired effects including weight loss and restricted growth in young *mdx* [15], and the efficacy of taurine in DMD patients remains to be investigated.

It has long been known that dystrophin deficiency leads to extensive muscle cell death both regulated (apoptosis) and unregulated (necrosis) in nature, with signs of the former preceding the latter [17]. Damage-Associated Molecular Patterns (DAMPs) released from dying cells [18] bind to Toll-like receptors (TLRs), which results in inflammasome formation [19]. In addition, the initial trigger of accidental necrosis may become amplified by loss of muscle fibers through regulated necrosis [20], a process that contributes substantially to the muscle wasting observed in the *mdx* [21]. Key regulators in this process termed necroptosis are the receptor-interacting Serine/Threonine protein kinases (RIPs) [22] of which RIP1 has a central role in the cell’s decision to live or die [23]. RIP1 is constitutively expressed in many tissues and becomes activated by pro-inflammatory signals. Tumor Necrosis Factor α (TNFα)-mediated cell death is a particularly salient feature of the initial stages of *mdx* pathogenesis. Interestingly, our former study evaluating taurine treatment in the earliest *mdx* disease phase observed significantly decreased mRNA levels of TNFα in tibialis anterior (TIB) muscle of taurine-treated *mdx* [24]. Binding of TNFα to its receptor TNFα Receptor 1 (TNFR1) is known to recruit RIP1 to the TNFR1 signaling complex, which leads to activation of the pro-inflammatory nuclear factor κB complex (NFκB). This shows how intimately necroptotic and inflammatory pathways are interwoven [25]. Coordinated interactions between RIP1 and RIP3 and the downstream effector protein Mixed Lineage Kinase domain-Like protein (MLKL) lead to the formation of a dedicated death complex called the necrosome that triggers necroptotic cell death. Additional mechanisms of skeletal muscle damage control are mediated by autophagy, a cellular process by which cellular components are degraded or recycled. Autophagosomes contain the lipidated membrane-bound form of microtubule-associated protein 1 light chain 3 (LC3 II) which interacts with sequestosome 1 (SQSTM1) to facilitate engulfment of ubiquitinated proteins and damaged organelles, and their degradation through fusion with lysosomes. Dysregulated autophagy within the muscle cells leads to muscle tissue degeneration [2].

In a preceding study, we evaluated amounts of healthy, regenerating, and necrotic muscle fibers in H&E sections from *mdx* tibialis anterior (TIB) muscle at 4, 8, 12, and 26 weeks of age, and observed the number of affected fibers to increase until eight weeks of age, later appearing to stabilize [26]. Active regeneration/degeneration of muscle fibers is an early event in *mdx* pathology [27] and these processes peaking past the 4-week timepoint may be attributable to the distinct conditions in our animal housing facility. In order to ascertain a timepoint at which significant induction of muscle fiber damage is assured in comparison to healthy mice, whilst still being early enough to capture this active disease phase, we selected the 5.5-week endpoint for our subsequent therapeutic studies. In *mdx* treated with 4.6 g taurine/kg body weight from birth until 5.5 weeks of age we observed improvements in skeletal muscle histology and a reduction in the expression of inflammatory cytokines TNFα and IL-1β and chemokine C-C motif ligand 2 (CCL2); however, these mice did not perform better in the hanging grid test evaluating stamina [24]. To further characterize taurine-induced effects, we now explored the levels and distribution of tissue restoration and cell death factors in *mdx* that were subjected to the same breeding conditions and taurine treatment regimen.

## 2. Materials and Methods

### 2.1. Mice

**Figure 1:**
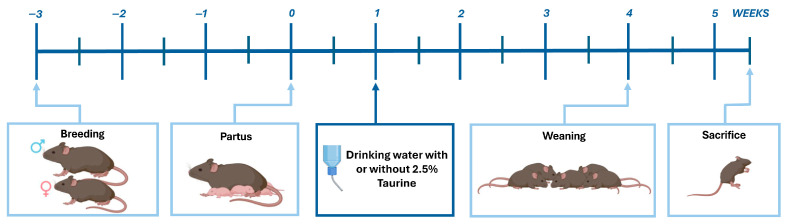
**Graphic representation of the treatment timeline.** Homozygous dystrophin-deficient C57BL/10ScSn-Dmdmdx/J (*mdx*) mice and wild-type C57BL/10SnJ control mice were bred by pairing males and females of the same genotype. The offspring was born approximately three weeks later. Taurine treatment commenced at postnatal day 7 via the drinking water consumed by lactating females. At four weeks of age, pups were weaned and continued to receive taurine through their own water supply. Mice were sacrificed at 5.5 weeks of age, and muscles were harvested for further analysis.

Dystrophin deficient C57BL/10ScSn-Dmdmdx/J (*mdx*) mice and the representative genetic healthy control strain C57BL/10SnJ fully checked for health were obtained at age 8 weeks (The Jackson Laboratory, Bar Harbor, ME, USA), after which they were bred and kept at the animal facility of Ghent University Hospital. Breeding conditions and experimental procedures adhered to international guidelines for animal experimentation and all procedures were approved by the local Animal Ethics Committee of Ghent University. All animals had access to food and water ad libitum. The disease phenotype of *mdx* is less severe than that of human disease. Consequently, homozygous *Dmd*^−/−^ mice are fertile and could be used for breeding in this study, thereby generating litters where males and females display DMD disease. In order to account for potential gender disparities, for instance those attributable to sex hormones that could influence the outcomes within the study groups, a balanced mix of males and females was employed in all experiments. Three groups were included in the experiment: untreated age-matched healthy controls (BLA, n = 8, one litter), *mdx* untreated controls (n = 8, one litter), and *mdx* receiving 2.5% taurine in the drinking water (n = 8, two litters). Throughout the experiment, the amount of water mice drank was monitored daily, except on weekends, by weighing the water bottle and dividing the weight by the number of mice. Water intake was similar between *mdx* mice receiving taurine (4.3 ± 0.1 mL per day) and control mice with access to regular drinking water (4.2 ± 0.2 mL per day). Groups contained both male and female mice in a 1 on 1 ratio. Taurine dosage corresponded to ≈4.6 g/kg body weight. The experiment was conducted in parallel for the three groups, and starting points were less than 3 days apart, minimizing confounding factors related to breeding/feeding conditions. Taurine treatment was initiated pre-weaning when pups were 1 week old, weaning was carried out at 4 weeks, mice were sacrificed at 5.5 weeks. General behavior was evaluated two-daily and all animals scored good, defined by no immobility, adequate grooming, and no excessive stress signals such as an arched back and erect hair on the back. In the *mdx* strain, special attention was paid that no dyspnea developed. At such a young age, disease burden of *mdx* did not reach unacceptable levels in any of the mice. Mice were anesthetized with a mixture containing 200 mg ketamine/mL (Dechra Pharmaceuticals, Northwich, UK) and 2% xylazine (Bayer, Leverkusen, Germany), and euthanized by cervical dislocation. Sets of three muscles were prelevated from the left hindlimb of individual animals: extensor digitorum longus (EDL), gastrocnemius (GAS), and TIB muscles. EDL and GAS muscles were frozen immediately on dry ice for Western blotting (WB), TIB was frozen in nitrogen-cooled isopentane for performing immunofluorescence (IF). The timeline for the treatment regime is shown in Figure 1.

### 2.2. Antibodies

Commercially available antibodies were used were rabbit polyclonal anti-RIP1 (Thermo Fisher Scientific, Waltham, MA, USA; WB, IF 2 µg/mL), rabbit polyclonal anti-RIP3 (Merck, Saint Louis, MO, USA; WB 2 µg/mL, IF 1.5 µg/mL), rabbit monoclonal anti-SQSTM1 clone D6M5X (Cell Signaling Technology, Danvers, MA, USA; WB, IF 1 µg/mL), rabbit monoclonal anti-LC3 II clone EPR18709 (Abcam, Cambridge, UK; WB 0.5 µg/mL, IF 0.75 µg/mL), rabbit monoclonal anti- glyceraldehyde-3-phosphate dehydrogenase (GAPDH) (Cell Signaling Technology; WB 0.5 µg/mL), rat monoclonal anti-F4/80 ab6640 (Abcam; IF 5 µg/mL), and goat polyclonal anti-CD56 (Bio-Techne R&D Systems, Minneapolis, MN, USA; IF 5 µg/mL).

### 2.3. Proteome Arrays

GAS muscle was ground in a glass–glass tissue grinder in an equal volume of extraction buffer (50 mM TrisHCl 2 mM EDTA pH 7.4) supplemented with complete mini EDTA-free protease inhibitor cocktail (Thermo Fisher Scientific). Samples were centrifuged at 10,000× *g* for 15 min at 4 °C, after which the supernatant was collected. The protein concentration was estimated based on the 260/280 nm absorbance using the Biodrop μLite device (Biochrom, Waterbeach, Cambridge, UK). An amount of 163 µg total protein from a female and a male mouse per group were pooled equally (2× 81.5 µg), generating 2 BLA control samples, 3 *mdx* control samples, and 3 taurine-treated *mdx* samples. Samples were loaded onto Mouse Apoptosis Proteome Profiler Arrays (Bio-Techne R&D Systems, Minneapolis, MN, USA), which were processed according to the manufacturer’s specifications. Relative levels of 21 apoptosis-related proteins were evaluated simultaneously on the nitrocellulose membranes via capture antibodies spotted in duplicates that were made visible through biotinylated detection antibodies and HRP-labeled streptavidin, and adding a chemiluminescent substrate. Spot intensity, captured by a Chemidoc device (Bio-Rad Laboratories, Hercules, CA, USA), corresponded to the relative amount of protein bound.

### 2.4. Western Blotting of Muscle Total Protein Fractions

GAS total protein extracts prepared as explained under 2.3 were diluted to a fixed protein concentration of 1500 μg/mL in lithium dodecyl sulfate buffer with reducing agent added (Thermo Fisher Scientific). Samples were boiled for 2 min, and 20 μL was loaded onto NuPAGE 10% bis-tris gels for electrophoresis in 3-(N-morpholino)propanesulfonic acid buffer (Thermo Fisher Scientific). The samples were then transferred to a nitrocellulose membrane via electroblotting. Membranes were subjected to a sequential Western blotting protocol, whereby the protein of interest was imaged first via chemiluminescent detection, and the housekeeping protein GAPDH was imaged subsequently using chromogenic detection on the same blot. The membrane was blocked for 1 h with milk blocking solution consisting of tris-buffered saline (TBS) and 2% non-fat dry milk (Bio-Rad Laboratories, Hercules, CA, USA). The membrane was washed repeatedly with TBS with 0.1% Tween 20 added (TBST), and incubated with primary antibodies in milk blocking solution overnight (see Section 2 for dilutions). The membranes were then washed with TBST and incubated with 0.5 µg/mL HRP-linked goat anti-rabbit IgG (Cell Signaling Technology) for 1 h. Membranes were again washed with TBST, after which protein bands were visualized using a chemiluminescent ECL Pierce substrate (Thermo Fisher Scientific) and imaged with the Chemidoc (Bio-Rad Laboratories). Subsequently, the same blot was subjected to GAPDH protein detection using the alkaline phosphatase-linked anti-rabbit Western Breeze kit, according to the manufacturer’s specifications (Thermo Fisher Scientific). Protein density quantification normalized to GAPDH content was carried out with Image-Lab 6.0 software (Bio-Rad Laboratories).

### 2.5. Western Blotting of Muscle Mitochondrial Protein Fractions

Mitochondrial protein extracts were prepared according to the protocol of Dimauro et al. [28] with some alterations. EDL muscles were homogenized in STM buffer (250 mM sucrose 50 mM tris-HCl 5 mM MgCl2 pH 7.4) supplemented with complete mini EDTA-free protease inhibitor cocktail (Thermo Fisher Scientific) and left on ice for 10 min. Samples were spun for 20 min 800× *g* at 4 °C, the supernatant was collected and spun for 20 min 11,000× *g* at 4 °C. The pellet was resuspended in STM buffer and spun for 10 min 11,000× *g* at 4 °C. The pellet was resuspended in SOL buffer (50 mM tris-HCl 1 mM EDTA 0.5% Triton-X-100 pH 6.8) and centrifuged for 10 min 800× *g* at 4 °C. The resulting supernatant contained the mitochondrial protein fraction. Protein concentration was measured using a Biodrop µLite spectrophotometer (Biochrom). Samples were supplemented with loading buffer containing 10% β-mercaptoethanol and 90% 4× Laemmli sample buffer, boiled for 2 min, and loaded in equal amounts of 30 µg protein onto 4–20% Mini-Protean TGX Stain-Free gels (Bio-Rad Laboratories). Proteins were transferred onto low-fluorescent polyvinylidene membranes pre-treated with ethanol by using the Trans-Blot Turbo system (Bio-Rad Laboratories). After protein transfer, blots were activated with the Chemidoc using Stain-Free blot pre-defined settings (Bio-Rad Laboratories). Blots were blocked in milk blocking solution for 1 h and incubated overnight with primary antibodies (see Section 2 for dilutions), 1 h with 0.5 µg/mL HRP-linked goat anti-rabbit IgG (Cell Signaling Technology), and visualized with ECL plus (Thermo Fisher Scientific) using the Chemidoc device (Bio-Rad Laboratories). Protein density quantification normalized to total protein was carried out with Image-Lab 6.0 software (Bio-Rad Laboratories).

### 2.6. Immunofluorescence

Eight µm frozen sections were cut from the middle part of TIB muscles. The sections were permeabilized for 2 min in cooled acetone, and incubated in phosphate-buffered saline (PBS) with 2% bovine serum albumin, 10% heat-inactivated human serum, and 5% donkey serum (PBS/BSA/HD) for 30 min. The primary antibodies were diluted in PBS/BSA/HD and incubated for 2 h. Sections were rinsed three times with PBS and incubated for 30 min with secondary antibodies: 0.25 µg/mL donkey anti-rabbit AlexaFluor 488 conjugated (Thermo Fisher Scientific), 0.12 µg/mL goat anti-rat AlexaFluor 488 conjugated (Thermo Fisher Scientific), 0.5 µg/mL donkey anti-rabbit CY3 conjugated (Jackson Laboratories, Bar Harbor, ME, USA), and 0.25 µg/mL donkey anti-goat CY3 conjugated (Jackson Laboratories) diluted in PBS. The sections were then rinsed three times with PBS and mounted with Fluoromount G (Thermo Fisher Scientific). Stained sections were visualized under a fluorescent microscope (Carl Zeiss, Jena, Germany) and recorded with a digital camera commanded by Cell^F Software version 2.4.112 (Olympus Life Science, Hamburg, Germany). Staining was evaluated by two independent researchers who were blinded to group allocation to allow objective scoring. Unblinded quantification of RIP1 fluorescence was performed with ImageJ version version1.54f (Rasband WS, National Institutes of Health, Bethesda, MD, USA). The region of interest of microscopic fields was selected to display complete tissue coverage without tissue edges or staining artifacts. Threshold levels were set to isolate RIP1 fluorescence in the green channel of three randomly selected 8-bit RGB images taken with 20× objective that originated from five untreated *mdx* mice and five *mdx* mice treated with taurine. All sections were stained on the same day and images were taken with fixed camera settings. Mean gray values were determined and reported as percentages, with 100% set at the mean level obtained in untreated *mdx*.

### 2.7. Statistical Analysis

Software G* Power version 3.1 (Heinrich Heine University, Duesseldorf, Germany) was used for sample size calculation, while earlier experiments had picked up taurine-induced effects in groups of 8 animals, confirming theoretical calculations. Quantifications were performed electronically, hence coding to treatment groups posed no problems of bias. A two-tailed *t*-test was performed to evaluate statistical significance. Normal distribution of the quantified protein levels and immunofluorescence were confirmed through a combination of graphical methods (histogram and quantile–quantile (Q–Q) plot) and the Shapiro–Wilk test, using SPSS Statistics software version 28 (IBM, Armonk, NY, USA).

All laboratory procedures were conducted at the Laboratory for Neuropathology of Ghent University, no part of the research was outsourced to external institutions.

## 3. Results

### 3.1. Protein Expression Levels

**Figure 2:**
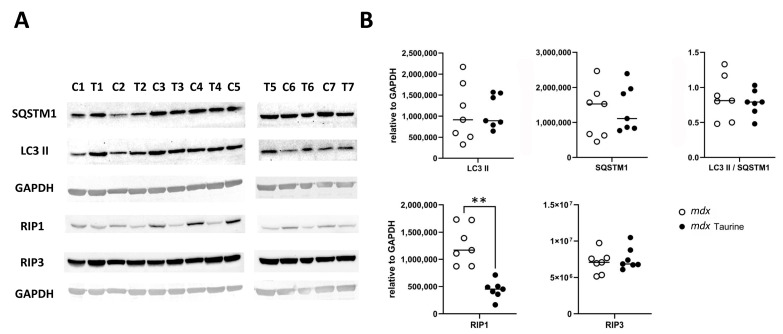
**Western blotting of total protein extracts from untreated and taurine-treated *mdx* mice.** (**A**) Protein bands representing sequestosome 1 (SQSTM1), lipidated microtubule-associated protein 1A/1B-light chain 3 (LC3 II), glyceraldehyde 3-phosphate dehydrogenase (GAPDH), receptor-interacting Serine/Threonine protein kinase 1 (RIP1), and RIP3 in total protein from the gastrocnemius muscle of seven untreated *mdx* controls (C1–C7) and seven taurine-treated *mdx* (T1–T7). Full blots are provided as Supplementary Figure S2. (**B**) Graphic representation of protein levels relative to GAPDH levels highlighting significantly reduced RIP1 levels (** *p* = 0.003) in taurine-treated compared to untreated *mdx*.

**Figure 3:**
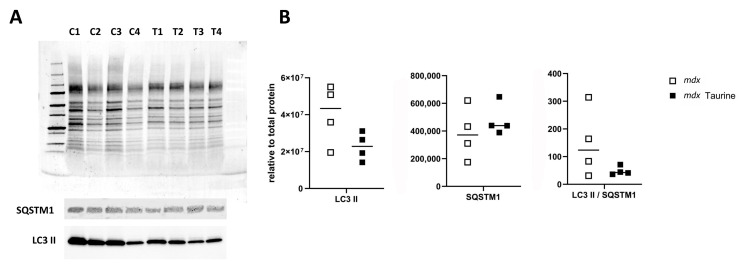
**Western blotting of mitochondrial protein extracts from untreated and taurine-treated *mdx* mice.** (**A**) Stain-free blot of mitochondrial protein fractions prepared from the extensor digitorium longus of four untreated *mdx* controls (C1–C4) and four taurine-treated *mdx* (T1–T4), and the protein bands representing sequestosome 1 (SQSTM1) and lipidated microtubule-associated protein 1A/1B-light chain 3 (LC3 II). Full blots are provided as Supplementary Figure S2. (**B**) Graphic representation of protein levels relative to total protein indicate no significant changes in LC3 II, SQSTM1 and their ratio between untreated and taurine-treated *mdx*.

Explorative protein arrays showed no overt differences between the levels of apoptotic proteins in pooled GAS muscle samples from the three mouse groups, being healthy control mice (BLA; n = 4), *mdx* (n = 6) and taurine-treated *mdx* (n = 6). The four most prominent proteins were cyclin-dependent kinase inhibitor 1B (p27 Kip1), Second Mitochondria-derived Activator of Caspases (SMAC)/Direct IAP-Binding protein with Low PI (Diablo), Heat Shock Protein (HSP)25/27, and HSP70 (Supplementary Figure S1). Based upon these observations, apoptotic proteins were not investigated further, and focus was placed on necroptotic and autophagic proteins. Protein bands were evaluated in total (Figure 2A) and mitochondrial (Figure 3A) muscle extracts using quantitative Western blotting. Significantly decreased RIP1 protein levels (*p* = 0.003) were observed in total protein extracts prepared from GAS muscle of taurine-treated *mdx* compared to untreated age-matched control *mdx*, while levels of RIP3 protein and LC3 II over SQSTM1 ratios remained unchanged (Figure 2B). The RIP1 effect size (equal groups, n = 7) was −2.937, with a confidence interval for d Cohen of −5.073 to −0.801 (computed for confidence coefficient 95%). The LC3 II/SQSTM1 ratio was also not significantly lower in mitochondrial protein extracts from EDL muscle protein extracts of taurine-treated *mdx* compared to untreated *mdx* (Figure 3B), pleading against a stimulatory effect of taurine on autophagy and mitophagy.

### 3.2. Protein Distribution in Muscle

**Figure 4:**
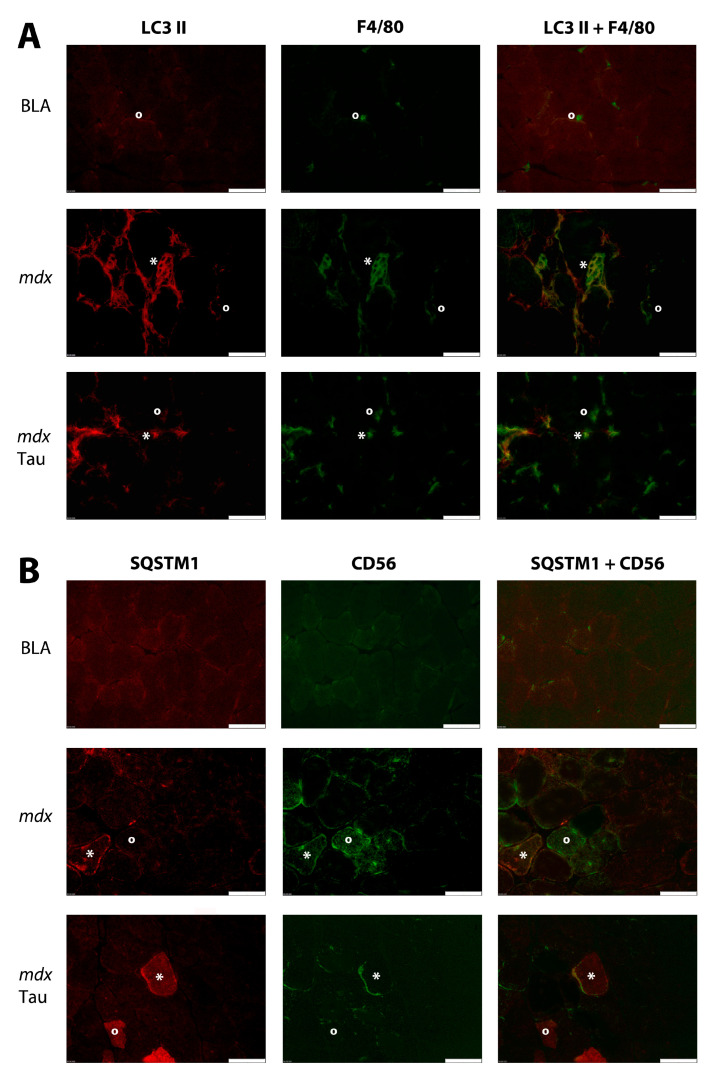
**Immunofluorescent staining for autophagic markers in mouse tibialis anterior muscle tissues.** Staining is shown in an age-matched healthy control (BLA), an untreated *mdx*, and a taurine-treated *mdx* (*mdx* Tau) mouse. (**A**) Double stainings for lipidated microtubule-associated protein 1A/1B-light chain 3 (LC3 II) (CY3, red) and the macrophage marker F4/80 (AlexaFluor488, green) are shown. (**B**) Double stainings for sequestosome 1 (SQSTM1) (CY3, red) and the marker for regenerating muscle fibers CD56 (AlexaFluor 488, green) are shown. A single positive (°) and double positive (*) muscle fiber have been marked. Scale bar = 50 µm.

**Figure 5:**
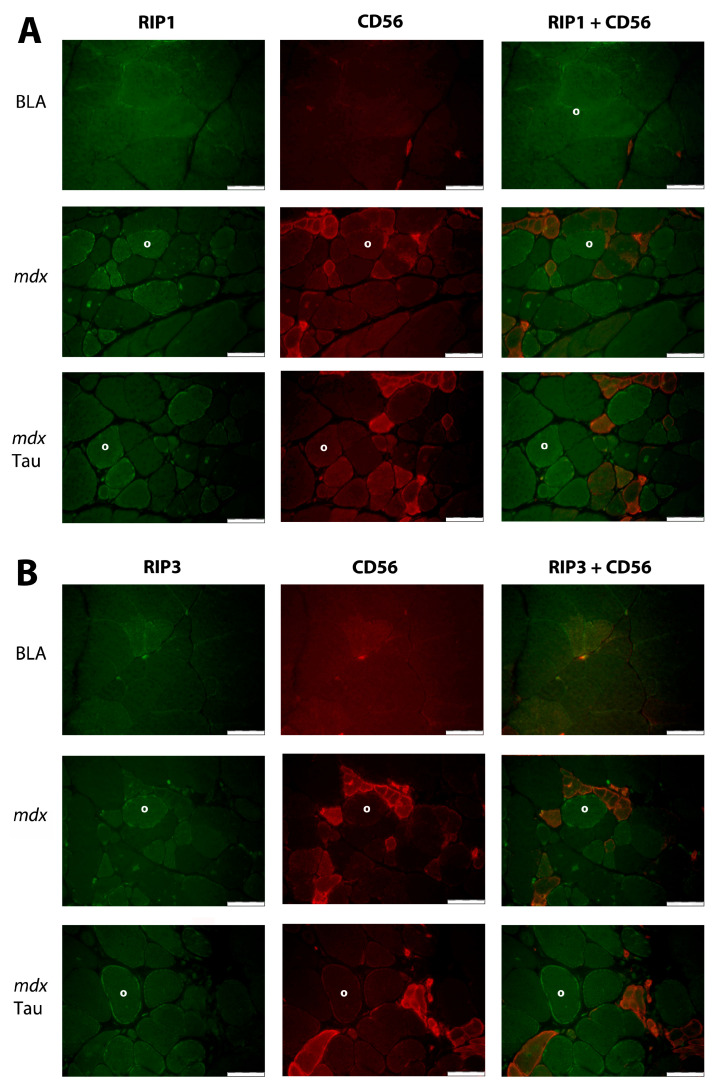
**Immunofluorescent staining for necroptotic markers in mouse tibialis anterior muscle tissues.** Staining is shown in an age-matched healthy control (BLA), an untreated *mdx*, and a taurine-treated *mdx* (*mdx* Tau) mouse. (**A**) Double stainings for receptor-interacting Serine/Threonine protein kinase 1 (RIP1) (AlexaFluor 488, green) and the marker for regenerating muscle fibers CD56 (CY3, red) are shown. Faint homogeneous muscle fiber staining pattern in BLA contrasts with a heterogeneous staining pattern in *mdx*, with strong RIP1 staining in subsets of muscle fibers of which the majority is CD56 negative. RIP1 staining remains patchy, but appears to diminish in taurine-treated *mdx*. (**B**) In BLA, faint receptor-interacting protein kinase 3 (RIP3) (AlexaFluor 488, green) staining in most and slightly increased staining in some muscle fibers is present. In *mdx*, a more heterogeneous staining pattern is observed, with stronger RIP3 staining in subsets of muscle fibers of which a majority is CD56 (CY3, red) negative. A RIP3 single positive (°) muscle fiber is indicated in each microscopic field. Scale bar = 50 µm.

Low homogeneous LC3 II staining was observed in muscle fibers without conspicuous differences between groups. The rare macrophages in BLA were LC3 II-negative or expressed low levels. In contrast, the majority of macrophages in *mdx* controls were LC3 II-positive, while in taurine-treated *mdx*, a smaller fraction of LC3 II-positive macrophages were present (Figure 4A). To objectivate this difference, macrophages were counted in eight microscopic fields from four individual mice of each group. The percentages of LC3 II-positive macrophages were 5% (1 of 21) in BLA, 70% (35 of 50) in control *mdx*, and 32% (29 of 90) in taurine-treated *mdx*. Low homogeneous SQSTM1 staining was observed in normal muscle fibers, which contrasted with the heterogeneous staining pattern in *mdx* muscle. Muscle fibers with intense SQSTM1 staining most often co-localized with CD56, a broad-spectrum cell marker that stains later-phase regenerating muscle fibers and immune cells. In taurine-treated *mdx*, both CD56 positive and negative muscle fibers were observed to display strong SQSTM1 staining (Figure 4B). A faint homogeneous muscle fiber RIP1 staining pattern was observed in BLA, contrasting with a heterogeneous staining pattern in *mdx*, with strong RIP1 staining in subsets of muscle fibers of which the majority were CD56 negative. RIP1 staining remained patchy, but appeared to diminish in taurine-treated *mdx* (Figure 5A). In order to confirm the observed trend, RIP1 staining intensity was quantified in randomly selected microscopic fields, and revealed a significant reduction (*p* < 0.0001) in taurine-treated *mdx* (80 ± 5%; n = 5) compared to untreated control *mdx* (100 ± 12%, n = 5) (Supplementary Figure S3). Similarly, RIP3 muscle fiber staining was faint in BLA, while *mdx* displayed a more heterogeneous muscle fiber staining pattern with stronger RIP3 staining in subsets of muscle fibers of which only a minority were CD56 positive (Figure 5B).

## 4. Discussion

### 4.1. Influence of Taurine on Muscle Cell Necroptosis

Necroptotic injury to the muscle tissues contributes to the pathogenesis of many muscle diseases including muscular dystrophies, and was first described in the *mdx* mouse model by Morgan et al. [21]. These authors reported a transient peak of RIP1 and RIP3 mRNA expression in TIB muscle of *mdx* at 3 weeks of age, coinciding with significantly higher RIP3 protein levels in TIB and GAS muscle [21], suggesting a transitory necroptotic peak in young *mdx*. Such regulated timeline of activation might represent a therapeutic window for interventions that could prevent necroptotic cell death kicking in. Elevated RIP3 expression has since also been observed in sartorius, biceps femoris, and diaphragm muscle of six-month-old Golden retriever muscular dystrophy dogs, in which levels correlated with increased myonecrosis [29]. Our study observed elevated levels of RIP1 and RIP3 in the *mdx*. Of these, RIP1 was selectively and significantly reduced in taurine-treated *mdx*. Taurine has been reported to regulate necroptosis by suppressing RIP1/RIP3/MLKL signaling in Klebsiella-infected mouse mammary epithelial cells [30,31] and liver cells exposed to valproic acid [32]. To our knowledge, this is the first report of taurine-induced changes in RIP1 levels in skeletal muscle tissue. Our findings suggest a potential additional beneficial effect in the *mdx*, in addition to the compound’s documented antioxidant and anti-inflammatory properties [16]. Similarly, the protective effect against TNFα-induced cell death observed for the antioxidant food additive butylated hydroxyanisole was additionally attributed to its ability to reduce RIP1 activation in cultured fibroblasts [33]. Immunofluorescent staining revealed faint homogeneous RIP1 and RIP3 muscle fiber staining in healthy age-matched control mice, contrasting with strong heterogeneous staining patterns observed in *mdx*. Increased RIP1 staining was not associated with muscle fiber regeneration, and for RIP3 staining also, only a subset of strong staining muscle fibers were identified as regenerating muscle fibers. In contrast to an earlier study [21], we did not observe an association between increased RIP3 staining and muscle fiber necrosis in the *mdx*. It should, however, be noted that percentages of necrotic fibers were very low in both groups, reaching 2.2 ± 0.3% in untreated and 1.9 ± 0.2% in taurine-treated *mdx*, respectively [24], possibly due to the young age of mice.

It has been demonstrated that necroptosis contributes to muscle damage in DMD. However, the prevailing notion that core necroptosis mediators are inherently detrimental to muscle recovery may warrant partial reevaluation. A surge in investigative activity has been observed, leading to the revelation of novel non-necroptotic functions of these factors. These functions include their regulatory influence on apoptosis and inflammatory responses [34]. The differential susceptibility of muscle cells to cell death signals is a subject that remains to be fully elucidated, due to the complexity of the mechanisms involved. The activation of necroptosis in muscle stem cells promotes muscle regeneration [35] and later switches off during myogenesis, with RIP1, RIP3, and MLKL levels significantly downregulated in myotubes [36]. Despite activated pro-apoptotic signaling, myotubes are, surprisingly, more resistant to apoptotic cell death than myoblasts, possibly due to enhanced anti-caspase mechanisms [37]. Intriguingly, RIP3^−/−^ mice additionally display RIP1 downregulation, seemingly without repercussions to skeletal muscle regeneration [29]. Necroptotic activation can, in turn, also occur through RIP1-independent activation of TLR signaling [38]. Overactive TLR pathways are involved in perpetuated muscle tissue damage in muscular dystrophies [39], and quenching TLR activities to appropriate levels for tissue maintenance represents a plausible therapeutic route for DMD. Moreover, the beneficial effects of necroptosis inhibition extend beyond skeletal muscle and also improve respiratory and cardiac phenotypes in *mdx* [29], which illustrates the broader cell-protective mechanisms that such therapeutic strategies could entail.

### 4.2. Influence of Taurine on Muscle Cell Apoptosis

Traditionally, myonuclear apoptosis has been described as a key early event in DMD-associated muscle degeneration [40]. Using apoptotic protein arrays, we could, however, not show any changes comparing young control mice with age-matched *mdx*, nor could we show any effects by taurine treatment. We observed abundant protein levels of p27 Kip1, SMAC/Diablo, HSP25/27, and HSP70 in GAS muscle, irrespective of mouse strain or therapy, pointing to their important general roles in muscle tissue growth and maintenance. p27 Kip1 and SMAC/Diablo are known regulators of muscle differentiation [41,42]; SMAC/Diablo commits to this function inside the mitochondria. This contrasts to the protein’s destructive function when it is released from the mitochondria [43], as is the case in reactive oxygen species (ROS)-induced muscle cell apoptosis [44]. The AMP-activated Protein Kinase (AMPK)/p27Kip1 pathway is a regulator of the autophagy/apoptosis balance in aged skeletal muscle cells, and was proposed as a target for improving muscle regeneration in older individuals [45]. Furthermore, it was demonstrated that AMPK activation enhances different disease-mitigating mechanisms in *mdx*, including amplified utrophin content and autophagy stimulation [46]. Hsp25/27 and Hsp70 are important factors for the buildup of muscle mass [47] and, therefore, their prominence in tissues from young mice is to be expected. In an earlier study, we reported age-associated differences in HSP70 protein levels, with a temporary upregulation of HSP70 in the earliest phase of *mdx* compared to age-matched healthy control mice [48]. The restorative activities of HSPs, which entail the refolding of proteins into their functional conformation, renders augmented expression a promising therapeutic strategy [49].

In light of the current body of knowledge regarding the complexity of regulated cell death, earlier studies describing abundant myonuclear apoptosis might need to be reexamined. Mechanisms distinct from the previously categorized dichotomous regulated (apoptosis) and uncontrolled (necrosis) cell death have since been identified, which reflect the true complexity displayed by cell death mechanisms [50]. In this regard, the first studies showing an involvement for ferroptosis [51] and pyroptosis [52] in the pathogenesis of DMD have been published, although elevated serum levels of growth differentiation factor-15 (GDF-15), a marker for pyroptosis [53], are not associated with DMD [54]. Intriguingly, taurine has recently been reported to reduce markers of ferroptosis in muscle cells both in vitro [55] and in vivo (in mice with muscle disuse atrophy) [56]. The osmolyte has also been credited with mitigating the effects of toxin-induced pyroptosis [57,58].

### 4.3. Influence of Taurine on Autophagy

Autophagy is a critical process in the regeneration of damaged muscle tissue and DMD is characterized by its dysfunction [59]. The levels of autophagic proteins LC3 and SQSTM1 display complex age- and tissue-dependent dysregulation in the *mdx* [60]. Taurine has repeatedly been shown to exert a positive effect on restorative autophagy [61]. TNF-treated L6 cells display increased LC3 II over LC3 I ratios, which can be normalized when taurine is added to the mix [62]. We could not show taurine to enhance the efficacy of autophagy, defined as the ratio of LC3 II to SQSTM1 protein expression levels, which was in contrast to the effect we observed with short-term treatment with the osmolyte ectoine [60]. DMD has also been associated with impaired autophagic removal of damaged mitochondria termed mitophagy [63,64]. Enhanced mitophagic capacity represents a potent booster of mitochondrial health and has been shown to mitigate the effects of aging [65]. Therefore, it is conceivable that this strategy could be explored for DMD. In support, taurine has been described as a mitophagy stimulator in various cell types [66]. A screen for genes upregulated by taurine in developing retinal cells has identified the long non-coding RNA taurine-upregulated gene 1 (Tug1) as essential for proper photoreceptor differentiation [67]. Tug1 expression has been observed to increase in human skeletal muscle in response to exercise and to regulate transcriptional networks associated with mitochondrial calcium handling, muscle differentiation, and myogenesis [68]. Our study, however, did not demonstrate significantly improved LC3 II over SQSTM1 protein ratios within the mitochondrial fraction of treated muscle tissue samples. Therefore, our results do not support the hypothesis that mitophagy is enhanced by short-term treatment with taurine.

Dysregulated innate and adaptive immune responses are hallmark pathogenic features of DMD [69]. The inflammatory processes downstream of dystrophin deficiency are an integral part of DMD pathology and, therefore, genuine therapeutic targets [70]. Chronic inflammation harming DMD muscle tissue is sustained by cytokines and chemokines abundantly recruiting inflammatory cells from the blood stream [71]. Copious amounts of macrophages are present in DMD skeletal muscle biopsies, with both pro-inflammatory inducible nitric oxide synthase (iNOS)-expressing M1 type and pro-tissue restorative CD206-expressing M2 type lineages represented. In the *mdx*, M1 predominate during the early acute stage, with the balance tipping over to the M2 phenotype in the regenerative and progressive later phase of the disease [72]. Macrophages allow muscle tissue remodeling via clearance of cellular debris through lysosomal trafficking involving LC3 II-marked organelles, canonical autophagy, and through LC3-associated phagocytosis, a process which is part of the innate immune response and becomes overactivated in DMD [73]. Taurine has been observed to antagonize M1 activation through the inhibition of macrophagic mitophagy [74], hence, the compound could aid to restrain the overzealous inflammation. It is noteworthy that the taurine transporter TauT/SLC6A6 undergoes upregulation during the polarization of macrophages to the M1 phenotype. It has been demonstrated that exposure to elevated taurine levels results in an increase in intracellular levels, leading to the inhibition of methionine metabolism and the subsequent blockage of PTEN-induced kinase 1 (PINK1)-mediated mitophagy. This hinders the conversion to glycolysis, which is an essential part of M1 functioning [75]. Our observation that the percentage of LC3 II-positive macrophages was reduced two-fold in taurine-treated *mdx* mice compared to untreated animals provides further evidence to support the hypothesis that taurine induces a reduction in the burden on the system for eliminating extracellular debris.

### 4.4. Consequences for DMD Therapy

Administering compounds that regulate necroptosis may yield beneficial effects in DMD. The results presented here demonstrate that taurine exerts a quenching effect on RIP1, a pivotal necroptotic regulator that has been posited as a potential therapeutic target for neurodegenerative and inflammatory diseases [76]. The study is, however, of an exploratory nature and has inherent limitations. First, more research is needed to confirm these findings and fully characterize taurine’s effects on the RIP1/RIP3/MLKL axis. In addition to quantifying proteins, it is necessary to investigate post-translational modifications, such as phosphorylation patterns, as they represent a potent additional layer of regulation. Second, mechanistic evidence should be obtained through in vitro and in vivo experiments, as well as through different disease models. The intricate interplay between the RIP1/RIP3/MLKL axis and other pathways remains to be fully elucidated. Importantly, evidence accumulates of taurine acting as an influencer of the differentiation of muscle cells from the immature myoblast to the functional multi-nucleate myotube stage. Muscle taurine concentrations are several times higher in fetal and neonatal periods, hence the suggestion that taurine may participate in muscle tissue buildup via processes that are regulated by the upstream phosphatidylinositol 3-kinase (PI3K)/protein kinase B (AKT)/mammalian target of rapamycin (mTOR) pathway [62]. Taurine’s undesired blunting effect on growth and muscle development, therefore, would need to be explored further. Third, the interactions of taurine with DMD standard of care need to be investigated, as combination therapies will remain the mainstay of DMD treatment for the foreseeable future. Fourth, the differential effects of taurine on the necroptotic pathway, related to timing and dosage, need to be investigated. The effects obtained may vary significantly between disease phases, such as the early phase of active degeneration and regeneration versus the late phase with established fibrosis and fat replacement. Furthermore, it is imperative to determine the therapeutic window and a realistic dose for a supplement in order to ascertain whether it can lead to phenotypic disease improvement without causing undue side effects. Finally, despite the fact that the genetic defect is comparable, the *mdx* disease phenotype is markedly less severe than human disease. Consequently, it is not possible to extrapolate the results obtained in preclinical studies of this disease model directly to patient management. A methodology that has been demonstrated to facilitate a more authentic simulation of the symptoms exhibited by human skeletal muscle in the *mdx* mouse model is the administration of a strenuous exercise regime. A proteomic study revealed that the aggravated phenotype exhibited by exercised *mdx* mice is associated with changes to the proteome residing in pathways involved in glucose metabolism, energy production and sarcomere structure. It is interesting to note that taurine treatment led to the reversion of some of these changes, suggesting that *mdx* may be better adapted to contraction-induced muscle tissue stress following treatment with taurine [77].

## 5. Conclusions

The maintenance of muscle tissue functionality is contingent upon a balance between destruction and preservation. Beneficial effects of taurine on the DMD mouse model *mdx* appear to be multifaceted and have been attributed to the osmolyte’s observed anti-inflammatory and anti-oxidative activities. This study is, to our knowledge, the first to add effects on necroptotic proteins to the list. We observed significantly reduced RIP1 protein levels in muscle tissues from *mdx* treated with 4.6 g taurine/kg body weight in the earliest disease phase, identifying the RIP1/RIP3/MLKL axis as an additional pathway worth further examination. The quenching effect elicited on RIP1 might contribute to the benefit of administering taurine; however, additional studies are needed to determine if a therapeutically meaningful effect could be achieved from optimal dosage and treatment timing.

## Data Availability

The data presented in this study are available on request from the corresponding author due to linkage of raw data to software that is not publicly available.

## Supplementary Materials

The following supporting information can be downloaded at: https://www.mdpi.com/article/10.3390/brainsci15111175/s1, Figure S1: Protein Arrays; Figure S2: Full Western blots; Figure S3: RIP1 immunofluorescence quantification.

## Abbreviations

The following abbreviations are used in this manuscript:

DMD	Duchenne muscular dystrophy
RIP1	Receptor-interacting Serine/Threonine protein kinase 1
AAV	Adeno-associated virus
ASO	Antisense oligonucleotide
DAMPs	Damage-Associate Molecular Patterns
TLRs	Toll-like receptors
RIPs	Receptor-interacting Serine/Threonine protein kinases
TNFα	Tumor Necrosis Factorα
TIB	Tibialis anterior
TNFR1	TNFα Receptor 1
NFκB	Nuclear factor κB complex
MLKL	Mixed Lineage Kinase domain-Like protein
LC3 II	Microtubule-associated protein 1 Light Chain 3
SQSTM1	Sequestosome 1
*mdx*	C57BL/10ScSn-Dmdmdx/J
BLA	Untreated age-matched healthy controls
WB	Western blotting
IF	Immunofluorescence
EDL	Extensor digitorum longus
GAS	Gastrocnemius
GAPDH	Glyceraldehyde-3-phosphate dehydrogenase
TBS	Tris-Buffered Saline
TBST	TBS with 0.1% Tween 20
PBS	Phosphate-buffered saline
Kip1	Cyclin-dependent kinase inhibitor 1B p27
SMAC	Second Mitochondria-derived Activator of Caspases
Diablo	Direct IAP-Binding protein with Low PI
HSP	Heat Shock Protein
TLR	Toll-like receptor
ROS	Reactive oxygen species
AMPK	AMP-activated Protein Kinase
GDF-15	Growth differentiation factor-15
Tug1	Taurine-upregulated gene 1
iNOS	Inducible nitric oxide synthase
PINK1	PTEN-induced kinase 1
