# Supplementary material for: Early Taurine Administration Decreases the Levels of Receptor-Interacting Serine/Threonine Protein Kinase 1 in the Duchenne Mouse Model mdx
Source: Brain Sci. 2025 Oct 30;15(11):1175. doi: 10.3390/brainsci15111175 (PMC12649910; doi:10.3390/brainsci15111175)
Supplement: Supplementary file 1 [file brainsci-15-01175-s001.zip › brainsci-3905823-supplementary.pdf]

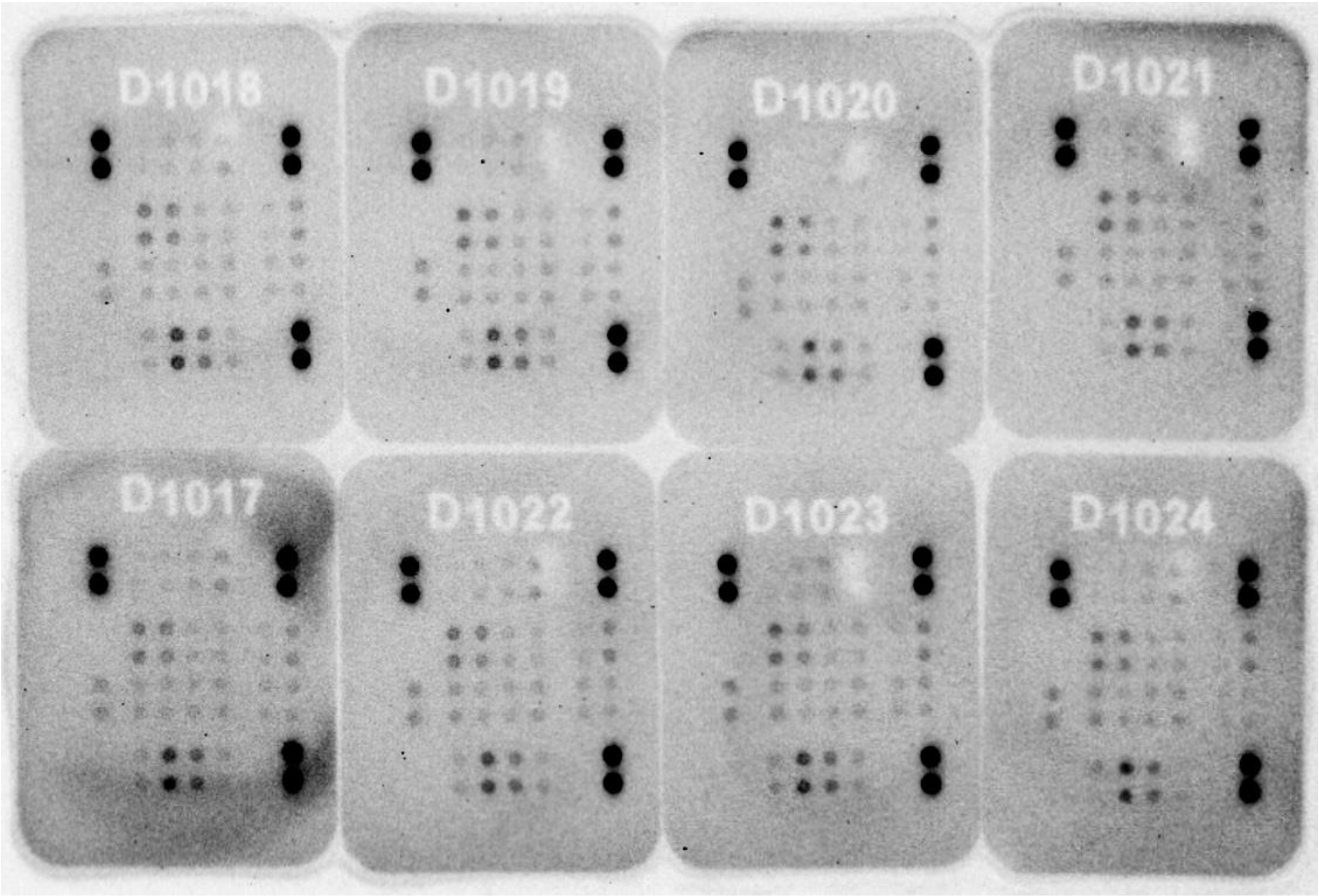

Figure S1: Protein arrays. Gastrocnemius muscle extracts containing 163µg total protein in samples pooled in equal amounts from a female and male mouse per group were loaded onto Mouse Apoptosis Proteome Profiler Arrays (bio-techne R&D Systems) processed according to the manufacturer's specifications. Duplicate spots representing 21 apoptosis-related proteins were captured simultaneously on nitrocellulose membranes by capture antibodies, biotinylated detection antibodies and HRP-labeled streptavidin and visualized with a chemiluminescent substrate using a Chemidoc device (Bio-Rad Laboratories). Protein arrays for healthy age-matched control samples (D1017-1018), untreated *mdx* samples (D1019-D021) and taurine-treated *mdx* samples from litter 1 (D1022) and litter 2 (D1023-D1024) show relative protein levels are similar between groups.

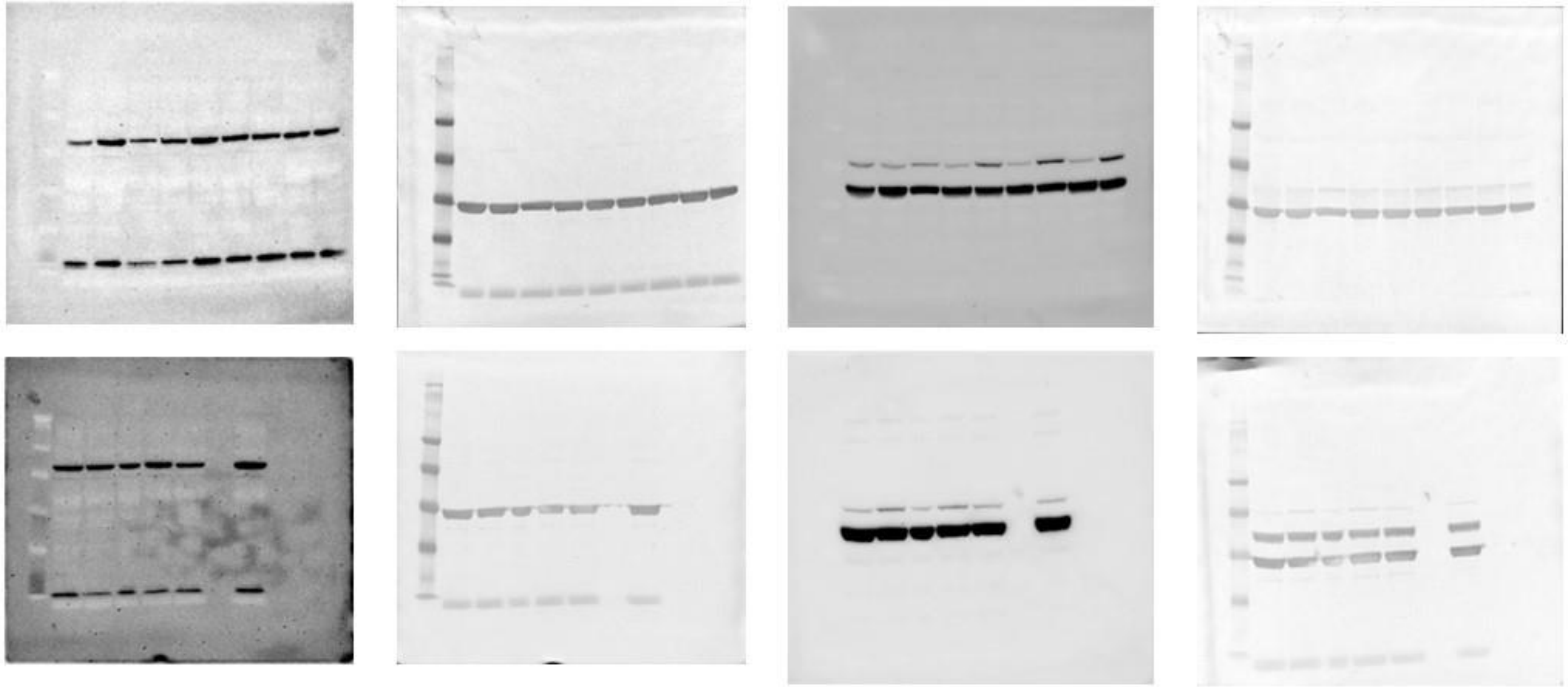

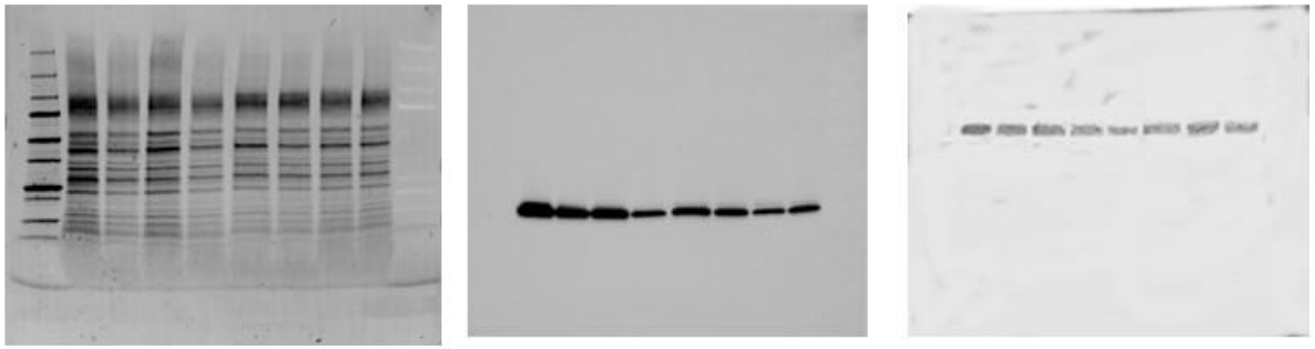

Figure S2: Full Western blots. Original and uncropped western blots are shown for protein bands represented in Figure 2 panel A and Figure 3 panel A.

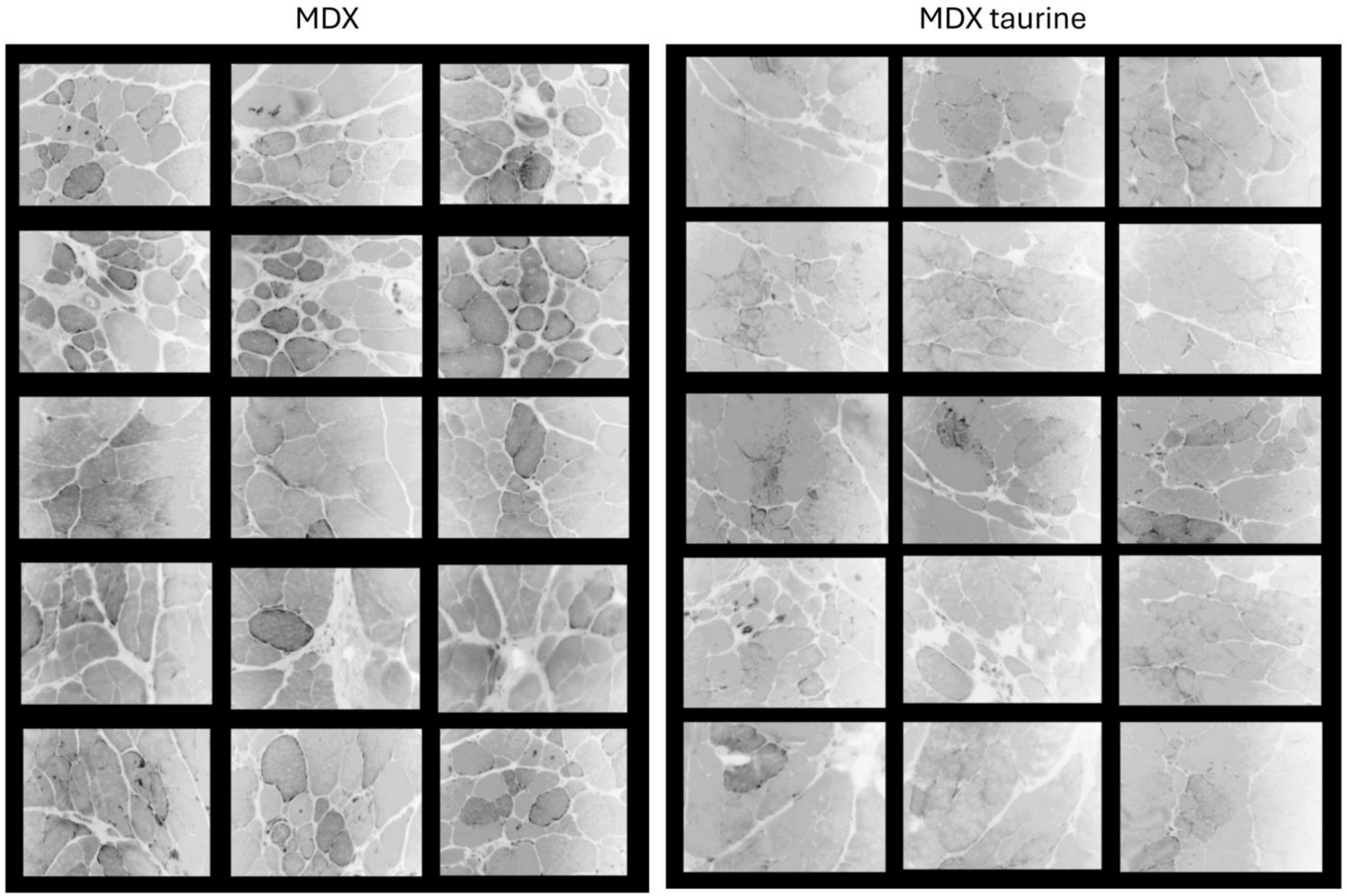

MDX	MDX taurine
21,68	22,96
26,96	22,26
33,28	23,38
29,52	22,77
30,14	24,77
31,73	21,18
30,36	22,59
31,07	21,59
28,8	23,66
29,57	20,8
25,61	19,48
27,43	22,05
27,66	23,25
23,33	24,82
23,51	22,89
**28.04±3.34**	**22.56±1.43**
**100±12%**	**80±5%**

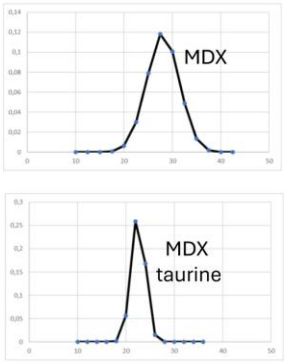

Figure S3: RIP1 immunofluorescence quantification. 8µm frozen sections from tibialis anterior muscle were immunostained with 2 µg/ml rabbit polyclonal anti-RIP1 and 0.25 µg/ml donkey anti-rabbit AlexaFluor 488 conjugated (Thermo Fisher Scientific) visualized under a fluorescent microscope (Carl Zeiss) and recorded with a digital camera using Cell F Software (Olympus Life Science). Quantification of RIP1 fluorescence was performed with ImageJ version 1.54f (Rasband WS) in three randomly selected microscopic fields per mouse originating from five untreated *mdx* mice and five *mdx* mice treated with taurine. Mean gray values were determined and are given as mean±SD and calculated as % compared to untreated *mdx*. Graphs show Gaussian distribution of values decreasing after taurine treatment.
